# Headache

**Published:** 1904-05

**Authors:** Chas. E. Boynton

**Affiliations:** Atlanta, Ga.


### HEADACHE.*

By CHAS. E. BOYNTON, M.D.,
ATLANTA, GA.

When I lightly accepted Dr. Stirling's invitation to read a paper here to-night on "Headache from all causes, except those due to

*Read before the Atlanta Society of Medicine on April 7, 1904.

the eye, nose, and alimentary canal," I little knew the difficulty of the proposition, and realizing my unpreparedness have unhesitatingly taken assistance from those more competent to handle the subject.

We must remember that "headache" is merely a symptom of many and varied disordered conditions, and can not claim the dignity of being a separate and distinct disease.

Yet this one symptom probably causes more suffering in the human race than any other single symptom, and will cause more frequent demands for relief than all others put together.

As a rule it is comparatively easy to cause a cessation of the pain temporarily by overpowering the sensory nervous system by the use of morphine, or easing it by the use of the coal-tar derivatives, but in many cases, unless the cause is removed, the pain returns as soon as the effect of the drug wears off, and the patient is really no better off than before. Hence it is very essential for us, so far as possible, to discover the cause and direct our efforts toward the origin of the trouble. The only way we can accomplish this is by considering each individual case on its own merits, and not leaving out any symptoms as unimportant. A carefully taken "previous history" and "present history," together with the "family history," often throw a good deal of light on the case under consideration. I think we are too apt when called to see a case of "headache" to simply try to give relief from the pain, and trust to luck that it will not return.

Headache covers a wide range in meaning, varying from a slight pain which is transient and not severe enough to much more than attract the attention of its host, to a condition which makes life itself almost unendurable while the pain lasts.

On looking into the *etiology* we find that all headache is either functional or organic in origin.

The *functional* class includes the vast majority of cases, while the cases of organic origin are much rarer, yet the prognosis as a rule is much worse.

Leaving out the conditions, which do not concern my part of the subject, we can include the *functional* cases under a few main headings. The first of these is:

*Toxemic.*—Headache which is due to the retention of toxic substances in the blood. Prominent in this class is the *nephritic headache* which takes either the uremic or congestive type.

In nephritic subjects *uremic headache* is quite common, and may be the only symptom of which the patient complains for a long time. Hence in any case of obscure, frequent or continued headache it is very important to examine the urine frequently to see if the kidneys are not at fault. With the granular kidney (except when it follows acute nephritis), the changes in the urine are often exceedingly gradual, and may escape the notice of the patient and even an ordinary analysis for albumin.

In these chronic cases, during the early stages, the urine is superabundant, there may be a trace of albumin or none, few casts or none, pale color, diminished specific gravity and a dimunition in the amount of urea and urates.

In the *later stages*, the albumin increases and the casts multiply, while the quantity is small with a very low sp. gr., and very little urea.

The headache, as well as the other nervous symptoms, are due to the retention of excrementitious substances in the blood, and nervous symptoms may be marked in any form of the disease either acute or chronic.

The headache may be the only nervous symptom for a long time, in chronic cases, but others are usually added before the end.

The pain may come on suddenly in the shape of neuralgic attacks and be intermittent, or may gradually increase in severity as the case progresses. The most frequent seat of the pain is the occipital region extending into the neck. Besides the headache there may be somnolence, nausea and vomiting, or also vertigo and optic neuritis simulating the general symptoms of tumor of the brain.

In the *exudative types* of kidney trouble, of course we have the œdema and the marked urinary changes to call attention to the cause of the headache.

The *congestive type* of nephritic headache is simply due to the disturbance of the cerebral circulation as brought about by the accompanying cirrhosis of the kidneys, alterio-sclerosis, and cardiac hypertrophy, together with the increased arterial tension.

*Puerperal Eclampsia.*—Fortunately this distressing condition often gives warning of its approach in the shape of premonitory symptoms, one of the most important of which is headache located in the frontal region or in the eyes and often accompanied by disturbance of vision.

This is of especial significance if œdema is present in any part of the body above the umbilicus, and especially if in addition there is sudden sharp pain of a colicky character in the epigastrium.

In these cases there may be albumin and casts in the urine, but there is always a diminution in the solids excreted and especially in the amount of urea.

The *next class* of toxic headaches is due to the products of *defective metabolism* and includes *rheumatism*, *gout* and *diabetes*.

*Rheumatism.*—In this disease we may have a headache which is simply due to the general toxic condition of the blood, or we may have a true myalgia of the head muscles, especially of the occipito-frontalis and its aponeurosis. In this case the pain will be increased by any movement of this muscle, either active or passive. Often there is a history of rheumatism, either muscular or articular, in other portions of the body. The attack is brought on by exposure and may last from a few hours to several weeks. It responds to antirheumatic treatment.

The *gouty* subject is frequently troubled with headache between the attacks of true gout. This headache may be due to the toxic condition of the blood or to disease of the arteries interfering with the cerebral circulation.

It is claimed that the headache and the vague symptoms known as lithæmia is merely an atypical form of gout. Fortunately the headache and other symptoms disappear with the appearance of the joint trouble.

Nervous symptoms are not uncommon in *diabetes mellitus*. Depression of spirits, weakness of mind, irritability of temper, and even melancholia with suicidal tendencies are only too often seen, and in addition some cases show symptoms resembling tumor of the brain, such as headache, vertigo, epileptic or apoplectic attacks and paralysis of the sixth pair of cranial nerves, or paralysis of a limb, etc.

*Infectious germs or their toxins,* are responsible for many of the headaches we meet in practice.

Almost every acute illness, especially if it is not inflammatory, is accompanied by marked headache, which is either frontal or diffuse.

In the early stage of typhoid fever or pneumonia, headache is often severe, persistent and accompanied by symptoms resembling those of meningitis and it may take several days to clear up the diagnosis.

The intense frontal headache of smallpox and influenza are both accompanied by intense aching in the back and limbs. In the febrile stage of malaria, headache is intense and sometimes is accompanied by delirium.

As a general rule headache occurs in every febrile case, and in any case of acute headache the temperature should always be taken.

*Various drugs* may produce headache which is toxic in origin.

*Alcohol* when taken in sufficiently large doses may cause a severe general headache of a congestive type, and when taken in large doses daily may produce a constant dull headache. This chronic headache is due to a combination of toxæmia, digestive disturbances and cerebral arteriosclerosis. Often there are cirrhotic changes in the liver and kidneys which give symptoms of their own.

The excessive use of *tobacco* may indirectly cause headache by producing digestive disturbances, changesin the heart's rhythm and cerebral circulation and finally by producing neurasthenia.

In some individuals *coffee* may be the cause of headache.

In *chronic lead poisoning* sometimes a severe headache accompanied by other cerebral symptoms is encountered and may lead to a fatal result.

The next and last variety of *toxic* headache is that which occurs in *Graves' disease* and is now thought to be due to autotoxæmia from hypersecretion of the thyroid gland. Headache is not a constant symptom in this condition, but it is sometimes very distressing and may be confined to one side of the head. It may be accompanied by a sense of throbbing in the head, a pulsating noise in the ears and transient vertigo.

Under *neuropathic conditions* we find three diseases which produce headache—neurasthenia, hysteria and epilepsy.

*Neurasthenic* patients often complain of headache, but it is not present in every case. The headache varies in intensity from a sensation of fullness or heaviness on the top of the head to a sensation as if an iron band was tightly constricting the head. The intense headache is more apt to follow mental exertion and quiets down after rest is taken.

The headache is usually slight in degree and is either continuous or occurs at brief intervals. It is not, as a rule, general, but is commonly located in the frontal or occipital region and occasionally accompanied by vertigo.

All of these sensations are modified by the amount of thought given them by the patient. The presence of other symptoms of neurasthenia make the diagnosis clear.

In *hysterical* patients the headache, as a rule, is similar to that of neurasthenia. It may be limited to one side, thus simulating migraine.

The peculiar character of the pain, its dependence upon the physical condition of the patient at the time, its rapid disappearance or variation in degree, the favorable influence of suggestion or diversion, together with other signs of hysteria—as paralysis, convulsions, disturbances of sensation, etc.—should make the diagnosis clear.

In obscure cases of *epilepsy* a diagnosis can sometimes be made by remembering that an attack of "*grand mal*" is often followed by a marked general headache which may last for several hours.

If a severe headache is present early in the morning, and is accompanied by soreness of the muscles and joints of the extremities, a bitten tongue or mucous membrane of the cheeks and a wet bed, a diagnosis of nocturnal epilepsy can be positively made.

Of the *reflex headaches* only two concern this part of the subject—uterine and sexual. When a patient suffering from uterine or ovarian disease is afflicted with headache, it should be considered merely as a symptom of neurasthenia, which is so frequent in women with intrapelvic disease. The headache is usually general and may be accompanied by tenderness of the scalp. The same may be said of affections of the male generative organs.

Headache may be caused by disease of the abdominal or thoracic organs. The head pain may be slight, but is always associated with tenderness of the scalp in certain areas. This scalp tenderness indicates a true *reflex headache.*

The *circulatory headaches* are due to disturbance of the cerebral circulation, producing either hyperæmia or anæmia of the brain tissue.

The hyperæmia is either active or passive.

*Active hyperœmia* means an increased arterial supply and is due to a paralysis of the vasoconstrictors, allowing distention of the vessels. In the majority of cases it is acute, and in severe cases is accompanied by a throbbing, pulsating headache, vertigo, tinnitus, insomnia, mental confusion and delirium, all of which are made worse by lowering the head.

The face and scalp are red and the conjunctivæ injected. There are throbbing of the temporal arteries and contracted pupil. The pulse is full and may be slow or increased in frequency. In some individuals such attacks occur periodically. The severer forms are rare, while milder cases are more common. The early stages of meningitis of the convexity resembles these cases.

The condition is usually due to mental overwork, emotional disturbances, alcoholism, excessive use of tobacco, onanism, and injuries to the head. It may follow sunstroke or sudden suppression of menses in plethoric women. Or it may be seen periodically in young women after the removal of the ovaries. These symptoms can be produced by nitrite of amyl, or nitroglycerin.

*Passive hyperœmia* is due to obstruction of the venous circulation, and is an important cause of habitual headache. The headache is dull and often accompanied by a fullness in the head. The face and ears may be slightly cyanotic. It is generally due to mechanical obstruction to the return of the blood from the head. It results from growths in the neck, heart disease, pulmonary emphysema, persistent coughs, etc. It may be caused by a tight collar. The headache is aggravated by straining at stool, coughing, sweeping, muscular effort, etc.

*Cerebral anœmia* is usually part of a general anæmia. Headache nearly always accompanies general anæmia whether it be due to chlorosis or to loss of blood.

The headache may be frontal, vertical or diffuse and is generally a dull ache and may be associated with attacks of syncope, dizziness and incapacity for mental work. The pupils are usually dilated. The headache often subsides when in the recumbent position with the head low down.

This brings us to the second part of the subject.

*Organic Headaches*, which are due to some pathological condition affecting either the exterior or the interior of the skull.

*Tumors of the Brain.*—The most common varieties are the sarcoma, tubercle, and gumma, and in later life carcinoma. The frequency of the different types varies with the age of the patient.

Persistent and intense headache with marked exacerbations is one of the most prominent symptoms of all types. Brain tumor without headache is rare.

In addition they suffer from vomiting (often without much nausea), which may be projectile. Convulsions, local or general, paræsthesia, vertigo, impaired vision, and later paralysis and blindness are often present. The severity of the pain is such that patients have been known to commit suicide to get relief. The headache may be general or it may be confined to the frontal, temporal or occipital region, changing its position from time to time. The location of the pain does not mean that the tumor lies underneath, for it may be in an entirely different position, and its location must be determined by localizing symptoms which are generally present. As a general rule the headache is much worse in those cases where the tumor is in the cerebellum or the convexity of the brain than when the base is involved. The pain is aggravated by everything which affects the cerebral circulation, such as mental excitement, alcohol, coffee, muscular exertion, coughing, etc.

*Cerebral Abscess.*—Headache, generally of great severeness, is one of the earliest and most constant symptoms of this condition. The headache, however, may simply be dull and persistent and referred to the location of the abscess.

As the abscess is developing, the pain at first may be slight and only occur periodically, but later the paroxysms become very marked. Everything which causes an increased flow of blood to the brain greatly increases the pain. The headache may be general, but more frequently it is at first confined to the seat of the abscess and later radiates over the head. Abscess in the cerebellum usually has the pain in the occipital region and in the neck.

Besides the headache there are usually other signs of intracranial pressure.

Fever is generally present but may disappear for a time and return higher than before. The most frequent cause of brain abscess is chronic disease of the middle ear.

*Acute Purulent Meningitis.*—This is nearly always a secondary process. The onset may be very sudden, with chill, high fever and severe symptoms from the start. In other cases indefinite perdromata exist for a time, but almost invariably it is the headache which directs the attention toward the nature of the trouble. This grows worse with more or less rapidity and generally becomes very violent. In some cases the pain varies considerably from hour to hour or from day to day. The location of the pain may be frontal, occipital or over the entire head. Symptoms in general continue to grow worse until the patient sinks into coma, but even then the headache continues, as shown by the patient continually raising the hand to the head and frowning whenever the head is moved.

In *inflammation of the dura* the headache is usually circumscribed, and is often accompanied by nausea or vomiting and slight fever. When only one side is involved, the pain is usually confined to that side.

*Intracranial Syphilis.*—As a general rule headache is the earliest, most constant, and for a long time may be the only symptom of intracranial syphilis.

The pain is apt to be dull in character with marked periodical exacerbations which are always worse at night, and at times it is almost unendurable. The pain is not circumscribed, unless the bone is diseased in one place, but is distributed over the frontal, parietal or temporal region and finally over the entire cranium. There is not much tenderness unless the bones are diseased. The other symptoms besides the headache vary according to the part of the brain involved.

*Mastoiditis and Sinus Thrombosis.*—Nearly all of these cases start from either disease of the middle ear or petrous portion of the temporal bone. Hence the pain starts in the ear and radiates over the side of the head on which the trouble lies. When the pain is especially severe it usually means that the dura is involved.

As the local and general pyæmic infection increases, the headache often diminishes, due to the deadening effect on the sensorium. Besides as a subdural, temporal or cerebellar abscess, or meningitis may develop, the symptoms of these conditions may be added.

*Diseases of the Cranial Bones, Periostitis.*—The majority of these cases are due to injury. In many cases, however, there may be periosteal nodes over the cranium. In these cases the headache is often constant and liable to be much worse at night. The diseased bone is tender on pressure. This periosteal headache of the cranium may be mistaken for that due to muscular rheumatism and vice versa.

The last type of headache to be considered is that due to *caries of the cervical vertebræ.* In caries of the upper cervical vertebræ occipital headache is quite common, and the pain may extend to the temporal and parietal regions. It is increased by movements of the head and by deep pressure over the vertebræ. The pain is usually accompanied by stiffness of the neck muscles and the head may be fixed in the median line.

There may be more or less deformity at the seat of the trouble.

The *diagnosis* of the cause of the headache in any given case can only be made by carefully considering the history and all of the accompanying symptoms. Too much stress should not be placed on the character of the pain or its location, for in all varieties these are liable to change from time to time.

The *treatment* of any case of headache depends entirely on the cause, hence the success of the treatment depends largely upon the correctness of the diagnosis.

In general it is a good plan in all cases to put them to bed on a light fluid diet, open the bowels with an active cathartic, flush the kidneys, and apply heat and counter–irritation, in the shape of mustard to the feet, wrists, etc., to draw the blood away from the head, except in anæmic cases. Very frequently, if the entire skin can be made warm by wrapping the patient in blankets and applying hot-water bottles to produce sweating, the headache will cease.

In addition the patient can be given any of the coal-tar derivatives, morphine, or any other headache remedy during the attack, and as soon as it is over every effort should be directed toward the cause of the trouble.

But after everything possible has been done, headaches will continue to trouble civilized humanity.

